# Socioeconomic, Demographic, and Environmental Determinants of Hemoglobin Levels Among Women: A Multilevel Analysis in South and Southeast Asia

**DOI:** 10.1155/ghe3/5430745

**Published:** 2026-06-07

**Authors:** Abdullah Al Islam, Bodrunnahar Barna, Md. Amjad Patwary, Mohammad Romel Bhuia, Md. Atiqul Islam

**Affiliations:** ^1^ Department of Statistics, Shahjalal University of Science and Technology, Sylhet, 3114, Bangladesh, sust.edu; ^2^ Department of Food Engineering and Tea Technology, Shahjalal University of Science and Technology, Sylhet, 3114, Bangladesh, sust.edu; ^3^ Department of Statistics, Jagannath University, Dhaka, Bangladesh, jagannathuniversity.org

**Keywords:** anemia, demographic health surveys, hemoglobin, multilevel model, risk factors, South and Southeast Asia

## Abstract

Anemia, mainly caused by hemoglobin (Hb) deficiency, is a serious health issue in countries that are developing, such as Bangladesh, India, Nepal, the Maldives, and Myanmar. This study examines the factors influencing Hb levels in women aged 15–49 across these nations using data from the Demographic Health Surveys (2011–2016). Analyzing 716,273 women from 71 subnational regions, we employed multilevel linear mixed‐effect modeling to identify key determinants. The study revealed that its mean Hb level was 117.5 g/L with a standard deviation (SD) of 16.5 g/L overall, with its highest mean in Nepal (123 g/L) with SD 15.4 g/L and the lowest in the Maldives (115.3 g/L) with SD 3.8 g/L. Anemia affected 51.3% of women, with the highest prevalence in the Maldives (58.5%) and the lowest in Nepal (40.6%). Regarding socioeconomic status and education level, Nepal exhibited the highest mean Hb levels among different socioeconomic groups, while the Maldives had the lowest. Linear mixed models identified several factors affecting Hb levels, including socioeconomic status, education level, contraceptive method, menstrual cycle, literacy, pregnancy, marriage, breastfeeding, amenorrhea, age, BMI, parity, access to water, and sanitation. Furthermore, a four‐level random intercept model proved more effective than other models in fitting the data. Our findings highlight the need for targeted interventions to reduce anemia rates in South and Southeast Asia, emphasizing socioeconomic, demographic, and environmental influences. These insights are crucial for policymakers and program managers aiming to enhance women’s health in these regions.

## 1. Introduction

Anemia, characterized by low hemoglobin (Hb) levels, is among the most severe global public health issues in the world, especially in developing countries [1]. Hb is an important protein that carries oxygen in the body and needs to be at the right level for a person to be healthy and fit. If Hb is below levels required by the body to provide cellular respiration and carry out other vital functions, signs and symptoms, such as weakness, fatigue, and poor mental function, are experienced. This iron deficiency anemia is a common type of anemia; it usually occurs more in women, creating challenges for women’s health and development in society at large [2].

Women in South and Southeast Asian countries, including Bangladesh, India, Nepal, the Maldives, and Myanmar, have anemia as a problem of great concern. While efforts have been made toward addressing this health burden, its prevalence is still dreadfully high in these regions. South and Southeast Asia display one of the world’s worst panoramas of anemia, with women of reproductive age being particularly vulnerable to this condition [3]. Thus, as per the Global Nutrition Report 2018, South and Southeast Asia contributed about 36% in the global burden of anemia among women in the reproductive age group [4]. Different socioeconomic, demographic, and environmental factors play a role in keeping the Hb levels low among women in South and Southeast Asia. These previous studies highlight the multifaceted nature of anemia prevalence and the risk factors associated with the region. The educational level of socioeconomic status is identified as an important determinant of anemia with women from lower socioeconomic backgrounds being at risk [5]. In addition, high levels of education correlate with lower rates of anemia [6]. Some services, such as prenatal care and iron supplementation programs, are also linked to the incidence of anemia in women in South and Southeast Asia. Poor access to healthcare facilities, especially in rural areas, aggravates the burden of anemia by delaying diagnosis and treatment [7]. Beyond this, dietary patterns also explain the prevalence of iron deficiency anemia among women in the region, including the poor consumption of iron‐rich foods and its low dietary diversity [8]. Factors, such as reproductive health near, menstrual cycle irregularities, pregnancy, and postpartum periods, exert various influences on women’s Hb levels across South and Southeast Asia. Nowadays, anemia through menstruation, combined with iron intake deficiency, may develop an iron deficiency among menstruating women [9]. Also, the pregnant woman faces an increased risk of developing anemia due to physiological changes during pregnancy that include increased iron demand and expansion of blood volume [10]. Such environmental factors as access to clean water and sanitation facilities also exhibit an influence on the anemia prevalence rate among women in South and Southeast Asia. Poor sanitation and hygiene practices contribute to the prevailing parasitic infections, such as hookworm infestations that aggravate iron deficiency anemia [11]. Besides, limited access to clean water resources undermined the ability of iron supplementation programs to work and also contributed to the burden of waterborne diseases that further exacerbate low Hb levels among women in the region [12]. Although there has been extensive research on anemia prevalence and determinants in South and Southeast Asia, more needs to be investigated to understand the interaction among these factors and contextualize the South and Southeast Asian countries and subnational regions. This study hopes to enrich this literature by identifying certain socioeconomic, demographic, and environmental factors affecting the Hb levels of women in South and Southeast Asia, determining the prevalence of anemia, assessing the association between several risk factors and Hb concentration, and evaluating the effects of interventions that addressed low Hb levels and anemia prevalence among women in the region. It is planned that with the implementation of evidence‐based interventions and policies, the region may take one step further toward decreasing the burden of anemia and improving women’s health status in South and Southeast Asia.

## 2. Methods

### 2.1. Study Design

The study investigated, using a cross‐sectional design, broad issues that are factors determining Hb levels in women among South and Southeast Asian nations. Data were gathered from recently available Demographic Health Surveys (DHS) conducted in Bangladesh (2011), India (2015–16), Nepal (2016), the Maldives (2016–17), and Myanmar (2015–16). These survey results provide nationally representative estimates of many health indicators, such as Hb levels, socioeconomic factors, and reproductive health characteristics. The DHS employs a stratified two‐stage cluster sampling design. Sampling weights provided in the DHS datasets were applied in the analysis to ensure nationally representative estimates.

### 2.2. Study Population

In this study population, women aged 15–49 years were selected for inclusion for analysis based on DHS surveys taken from the five South and Southeast Asian countries. The study was limited to women who had their Hb levels measured during these surveys. The total estimated population of interest consisted of 716,273 women across the five countries. Table 1 contains details about the data sources for all surveys that were included in the analysis.

**TABLE 1:** The sources of data for each of the included surveys.

Survey	Year	Sampling design	Sample size	Women with Hb data
Bangladesh Demographic and Health Survey (BDHS)	2011	Two‐stage stratified sampling design	17,141 households	5684
India National Family Health Survey (NFHS‐4)	2015–16	Nationally representative survey with multistage sampling	628,892 households	684,833
Nepal Demographic and Health Survey (NDHS)	2016	Two‐stage stratified sampling design	11,040 households	6403
Maldives Demographic and Health Survey (MDHS)	2016–17	Two‐stage stratified sampling design	6050 households	6852
Myanmar Demographic and Health Survey (MMDHS)	2015–16	Stratified two‐stage sample design	13,206 households	12,501

### 2.3. Study Variables

#### 2.3.1. Dependent Variable

The primary outcome variable of interest was the Hb level, measured in grams per deciliter (g/L). Anemia was defined as a Hb level below 120 g/L, with severity categorized as mild (Hb: 100–119 g/L), moderate (Hb: 70–99 g/L), or severe (Hb: < 70 g/L) based on established thresholds [13].

#### 2.3.2. Independent Variable

This study is regarded as a comprehensive set of socioeconomic, demographic, and environmental variables as independent variables. These variables were recorded at various levels, including individual, household, cluster, regional, or national levels. A detailed description of the independent variables used in the analysis is provided in Table 2.

**TABLE 2:** Descriptions of independent variables used in this study.

Variables	Description
Country	1 = Bangladesh
	2 = India
	3 = Nepal
	4 = Maldives
	5 = Myanmar

Place of residence	1 = Urban
	2 = Rural

Women who are married	0 = No
	1 = Yes

Socioeconomic status	1 = Poor
	2 = Middle
	3 = Rich

Literacy	0 = No
	1 = Yes

Highest education level	0 = No education
	1 = Primary
	2 = Secondary
	3 = Higher

Currently pregnant	0 = No or unsure
	1 = Yes

Currently breastfeeding	0 = No
	1 = Yes

Currently amenorrheic	0 = No
	1 = Yes

Current contraceptive method	0 = Not using
	1 = Pill
	2 = IUD
	3 = Others

Menstrual period	1 = Regular cycle (last menstruation max. 6 weeks ago)
	2 = last time 6 weeks to 6 months ago
	3 = last time to 6 months to 1 year ago
	4 = more than 1 year ago
	5 = In menopause/hysterectomy
	6 = Before last birth
	7 = Never menstruated

Tap water	0 = No
	1 = Yes

Access to sanitary toilet	0 = No
	1 = Yes

Mother age	A continuous variable

BMI	A continuous variable

No. of children born	A continuous variable

### 2.4. Analysis of Statistical Data

Descriptive statistical methods were applied to summarize the data, with categorical variables expressed as percentages and continuous variables reported as mean ± SD or median ± IQR, based on their distribution characteristics. To determine variables associated with risk, a multilevel modeling technique was used. Multilevel regression analysis, using linear mixed models (LMM), was performed to account for explanatory variables across different hierarchical levels of the DHS data. The data followed a four‐level nested structure: Mothers/individuals (level 1) were nested within clusters or primary sampling units (level 2), clusters were nested within administrative regions or divisions or provinces (level 3), and regions were nested within countries (level 4). This hierarchical structure reflects the DHS sampling design and allows for appropriate adjustment of correlated observations within the same geographic and administrative units. Mothers residing in the same cluster, region, or country may share similar socioeconomic, environmental, and health‐related characteristics, which can induce intragroup correlation. Random intercepts were therefore included at the cluster, region, and country levels to capture unobserved heterogeneity operating at each level. The region‐level random effect accounts for subnational variation (e.g., divisions or provinces), reflecting differences in local infrastructure, health service accessibility, environmental conditions, and socioeconomic development. The country‐level random effect captures broader national differences, including variations in health systems, policies, economic conditions, and nutritional programs. The analysis addressed the challenge of correlated Hb levels within clusters, regions, and countries by incorporating random effects in regression models. Both univariate and multivariable linear mixed‐effects models were employed. A univariate mixed model was performed to investigate associations between individual predictors and Hb levels. Risk factors for low maternal Hb concentration were assessed using likelihood ratio–based Type 3 tests [14]. The LMM extends the general linear model, assuming a linear relationship between factors, covariates, and the dependent variable. Categorical predictors are chosen as factors, where each level may have a different linear effect on the dependent variable. Factors can be either fixed‐effects, representing variables with values of interest represented in the data, or random‐effects, reflecting variables sampled randomly from a larger population and useful for explaining excess variability. Scale predictors serve as covariates, assumed to have linear correlation with the dependent variable within combinations of factor levels.

Suppose *Y*_*i* *j* *k* *l*_ was the Hb level (continuous response variable) for the *l* ^th^ mother belongs to *k* ^th^ cluster, *j* ^th^ region of the *i* ^th^ country. It was assumed that the index follows a four‐level model as follows:

(1) Yijkl=XijklTβ+ηi+µij+αijk+εijkl,

where $X_{ijkl}^{T}$ = vector of explanatory information. *β* = vector of the regression parameter. *η*_*i*_ = country‐specific random effect. *µ*_*i* *j* _ = region‐specific random effect. *α*_*i* *j* *k*_ = cluster‐specific random effect. *ε*_*i* *j* *k* *l*_ = mother‐specific random effect.

It was assumed that the level‐specific random effects were identically and independently distributed with mean zero and homoscedastic random‐effect variances $\sigma_{ƞ\left(\text{country}\right)}^{\wedge 2}$, $\sigma_{µ\left(\text{region}\right)}^{\wedge 2}$, $\sigma_{\alpha \left(\text{cluster}\right)}^{\wedge 2}$, and $\sigma_{\varepsilon \left(\text{residual}\right)}^{\wedge 2}$, respectively. Assuming normality of the level‐specific random effects, the model can be developed by the following standard maximum likelihood (ML) or restricted ML (REML) method. In this analysis, the REML method was used to estimate the parameters. Multilevel models were fitted sequentially to account for the hierarchical structure of the data. Model labels reflect the levels included: C = country, R = region, and CL = cluster. Thus, 2L.C represents a two‐level model with country‐level random effects; 3L.R.C includes random effects at three levels at the region and country levels; and 4L.CL.R.C includes random effects at four levels at cluster, region, and country levels.

### 2.5. Model Selection Criteria

Several criteria were considered for model selection, including corrected Akaike’s information criterion (AICc), Bayesian information criterion (BIC), likelihood ratio test (LRT), and intracluster correlation (ICC). Generalized least squares estimation maximized the log‐likelihood function to estimate fixed‐effects parameters, yielding best linear unbiased estimators (BLUE) for fixed‐effects and best linear unbiased predictors (BLUPs) for random effects. Data analysis was conducted using the R language (Version 3.6.0), and SPSS Version 22.0 was employed for descriptive analysis, graphical representation, and data management.

## 3. Results

The major risk factors of women’s Hb levels were investigated, and variations in Hb outcomes across different levels were studied. This study was deemed necessary due to the persistent prevalence of anemia in South and Southeast Asian countries. Five countries, Bangladesh, India, Nepal, the Maldives, and Myanmar, were considered, and a continued examination of low Hb levels and associated risk factors was conducted using a LMM. Furthermore, investigation of cluster‐level, region‐level, and country‐level differences in women’s Hb levels allowed for the identification of any unanswered questions regarding variations in Hb levels. Hierarchical data structures were present in DHS data, where women aged 15–49 years (level 1) were nested within a cluster (level 2), clusters were nested within a region (level 3), and regions were nested within a country (level 4). Failure to recognize the hierarchical structure of the data could have led to underestimation of the standard errors (SE) for the regression coefficients, potentially resulting in spurious statistical significance and incorrect inference. Therefore, analyzing these data to determine the risk factors of mother’s Hb levels required the application of multilevel modeling to account for their hierarchical structure.

### 3.1. Descriptive Analysis

#### 3.1.1. Hb Level According to Country

**FIGURE 1:**
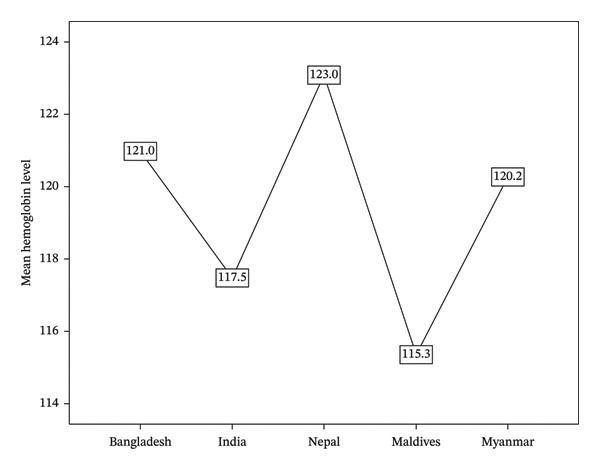
Estimated mean Hb level of women aged 15–49 years by country level.

The aggregated dataset included data of 716,273 women whose age was 15–49 years. The mean Hb level across the dataset was 117.5 g/L, with a standard deviation (SD) of 16.5. Hb levels showed notable differences between countries. Figure 1 presents a line chart illustrating the mean Hb levels by country. Among the countries, Nepal reported the highest mean Hb level at 123 g/L, while the Maldives recorded the lowest at 115.3 g/L. Bangladesh and Myanmar demonstrated relatively higher mean levels at 121 g/L and 120 g/L, respectively. India’s mean Hb level was 117.5 g/L, closely aligning with the overall mean.

The regional distribution of mean Hb levels is provided in Supporting Table 1, covering 71 divisions/regions/provinces across the five countries. In Bangladesh, the Barisal division recorded the lowest mean Hb level of 119.6 g/L compared to other divisions. India, with 36 regions, saw the Dadra and Nagar Haveli region exhibiting the lowest mean Hb level of 108.5 g/L, highlighting a significant burden of anemia among women. Among six regions, the Central Region recorded the lowest mean Hb level of 110.8 g/L in the Maldives. Similarly, Myanmar and Nepal, with 15 and seven divisions/regions, respectively, reported mean Hb levels of 116.5 g/L in the Magway region and 115.6 g/L in Province 2, signifying variations within their respective regions.

#### 3.1.2. Anemia Prevalence by Country

**FIGURE 2:**
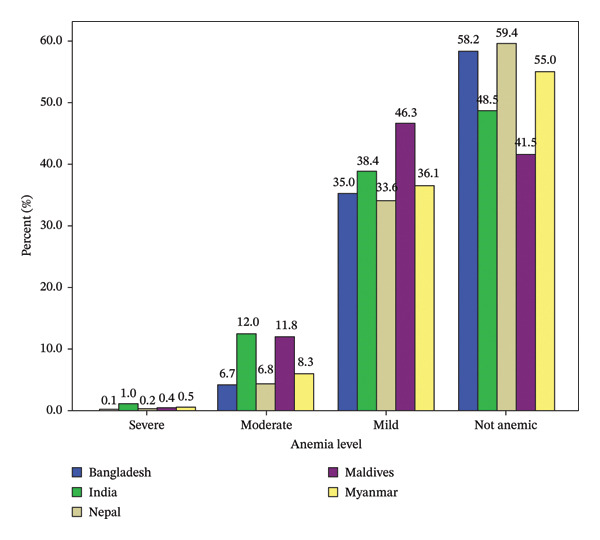
Estimated prevalence of anemia in women aged 15–49 years by country level.

The overall prevalence of anemia among women was 51.3%, with 38.4% classified as mild, 11.9% as moderate, and 1% as severe. Among the five countries, the Maldives had the highest prevalence of anemia at 58.5%, while Nepal had the lowest at 40.6%. Figure 2 illustrates the distribution of the three types of anemia levels and nonanemic individuals across the countries. In terms of country‐level prevalence, the Maldives exhibited the highest prevalence of mild anemia at 46.3%. India, however, had the highest prevalence of moderate (12.0%) and severe (1.0%) anemia, with the prevalence of mild anemia ranking second highest at 38.4% compared to other countries.

#### 3.1.3. Mean Hb Levels Across Countries According to Socioeconomic Status

**FIGURE 3:**
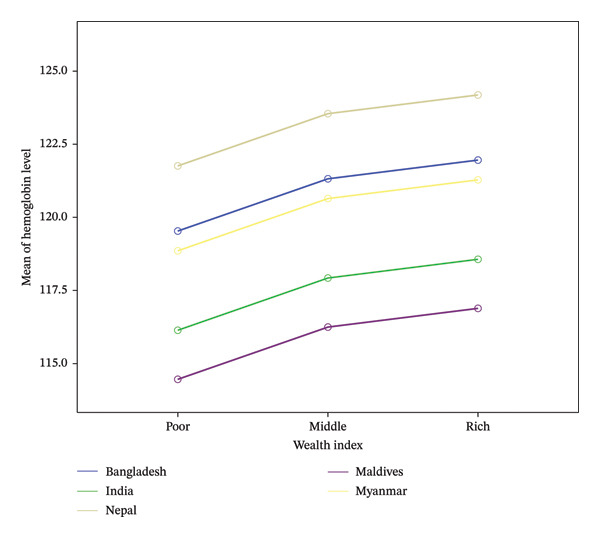
Trend in mean Hb level of women aged 15–49 years among countries by socioeconomic status.

The household socioeconomic status serves as a crucial indicator, reflecting a household’s overall economic well‐being, aligning with measures of expenditure and income. It serves as a comprehensive measure of a household’s overall living quality. Figure 3 illustrates a pattern in the mean Hb levels among women in five countries at various levels of the socioeconomic status. Across all countries, there is a noticeable upward trend in Hb levels with increasing socioeconomic status. Notably, Nepal shows the most favorable scenario, with higher Hb levels observed across all socioeconomic status categories compared to other countries. However, Hb levels in the Maldives were lower among poor, middle, and rich families compared to those in the other nations.

#### 3.1.4. Mean Hb Level Among Countries According to Mother’s Education

**FIGURE 4:**
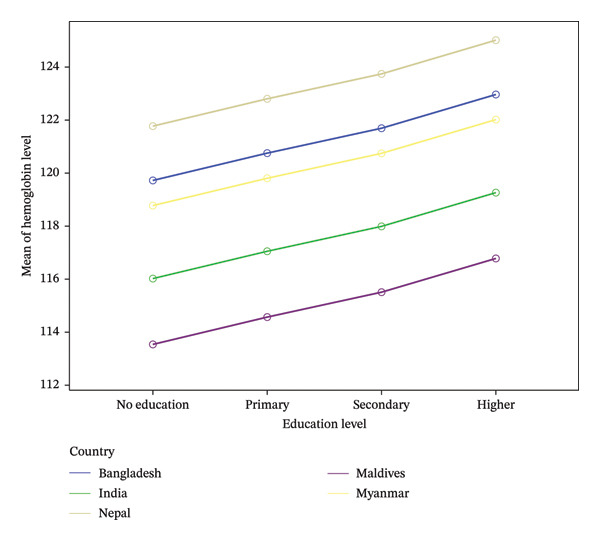
Trend in mean Hb level of women aged 15–49 years among countries by education level.

Women’s education serves as a crucial indicator of their Hb levels and reflects the level of socioeconomic development. It is a significant determinant of individuals’ knowledge, attitudes, and behaviors. Figure 4 illustrates the trend in mean Hb levels among women across countries based on their education level. The data reveal that Hb levels increase with higher levels of education in each country. However, in the Maldives, Hb levels are consistently lower across all education levels compared to other countries. In contrast, Nepal exhibits the most promising scenario, with a notably steeper increase in Hb levels across education levels compared to other countries.

#### 3.1.5. Sociodemographic Characteristics of Hb Level

The women in the study had a mean age of 29.89 years (SD: 9.76), and the average age at first delivery was 20.54 years (SD: 3.84), and on average, they had 1.89 children (SD: 1.83) by their reproductive age, with households typically consisting of 5.79 members (SD: 2.66), and the mean body mass index (BMI) was 2176.1 (SD: 417.52) (refer to Supporting Table 2). Supporting Table 3 displays the characteristics of associated factors based on Hb levels. Utilizing a LMM, all factors were found to be statistically significant with Hb levels. Notably, the mean Hb level was lowest in the Maldives at 115.34 g/L (SD: 13.75) and highest in Nepal at 123.05 g/L (SD: 15.34), with both differences being statistically significant (*p* < 0.0001). Bangladesh and Myanmar exhibited nearly equal mean Hb levels at 120.95 g/L and 120.25 g/L, respectively. India had the second‐lowest mean Hb level at 117.45 g/L. Regarding residence, women in urban areas had significantly higher Hb levels (118.42 g/L) compared to those in rural areas (117.20 g/L). Other factors, such as marital status, current employment, socioeconomic status, literacy, education level, pregnancy, breastfeeding, amenorrhea, contraceptive methods, menstrual period, husband/partner’s educational level, husband/partner’s occupation, tap water, and access to sanitary toilets, were also found to be significant predictors of Hb levels.

### 3.2. Univariate Analysis

The study aimed to explore variations in women’s Hb levels (level 1) across different levels of risk factors, including clusters (level 2), regions (level 3), and countries (level 4). To assess the impact of associated risk factors on Hb levels of women aged 15–49 years, a LMM was employed at the cluster, regional, and country levels. Initially, each covariate was examined using a univariate LMM to measure its significance. Variables with a *p* value < 0.10 in the Type 3 test were considered candidate variables for the full model selection process. Table 3 illustrates the results of the univariate analysis of the four‐level LMM, indicating significant associations between all considered factors and women’s Hb levels.

**TABLE 3:** Test of the univariate four‐level model for individual characteristics.

Variable name	Estimate	SE	*p* value
*Place of residence*			
Urban^∗^			
Rural	−1.02	0.08	< 0.0001

*Married women*			
No^∗^			
Yes	−0.56	0.04	< 0.0001

*Socioeconomic status*			
Poor^∗^			
Middle	1.03	0.05	< 0.0001
Rich	2.03	0.05	< 0.0001

*Literacy*			
No^∗^			
Yes	0.79	0.04	< 0.0001

*Highest educational level*			
No education^∗^			
Primary	0.23	0.06	0.0002
Secondary	0.63	0.05	< 0.0001
Higher	1.71	0.07	< 0.0001

*Currently pregnant*			
No or unsure^∗^			
Yes	−8.34	0.09	< 0.0001

*Currently breastfeeding*			
No^∗^			
Yes	−1.49	0.05	< 0.0001

*Currently amenorrheic*			
No^∗^			
Yes	−1.55	0.08	< 0.0001

*Current contraceptive method*			
Not using^∗^			
Pill	3.14	0.11	< 0.0001
IUD	−0.57	0.16	0.0004
Others	0.87	0.04	< 0.0001

*Menstrual period*			
Regular cycle (last menstruation max. 6 weeks ago)^∗^			
Last time 6 weeks to 6 months ago	−1.72	0.06	< 0.0001
Last time to 6 months to 1 year ago	−5.53	0.10	< 0.0001
More than 1 year ago	1.51	0.16	< 0.0001
In menopause/hysterectomy	2.48	0.08	< 0.0001
Before last birth	−2.06	0.10	< 0.0001
Never menstruated	0.49	0.24	0.039

*Tap water*			
No^∗^			
Yes	0.49	0.05	< 0.0001

*Access to sanitary toilet*			
No^∗^			
Yes	1.39	0.05	< 0.0001

Mother age	0.03	0.002	< 0.0001

Body mass index	0.004	0.00005	< 0.0001

No. of children born	−0.05	0.01	< 0.0001

Number of household members	−0.03	0.007	< 0.0001

^∗^Reference category.

### 3.3. Exploring the Association Among Risk Factors and Hb Levels Using a Multilevel LMM

In the univariate analysis, all identified potential risk features were included in the entire model applying multilevel LMMs. Subsequently, a backward elimination procedure was applied to develop the final model, accounting for the correlations between cluster‐specific effects (level 2), region‐specific effects (level 3), and country‐specific effects (level 4). During model selection, the models were compared based on AICc, BIC values, and LRTs with the final model selected based on the smallest values of AICc and BIC. Variables with a *p* value < 0.05 were considered significant risk factors for Hb levels. Several multilevel models were developed incorporating mother‐, cluster‐, region‐, and country‐specific hierarchies for Hb levels. These included 2‐level at country‐specific, 3‐level at region‐ and country‐specific, and 4‐level at cluster‐, region‐, and country‐specific models, denoted as 2L.C, 3L.R.C, and 4L.CL.R.C, respectively. The summary statistics provided in Table 4 indicate that the 4‐level country‐specific model outperformed the 3‐level and 2‐level models based on AICc, BIC, and LRT. In both comparisons, *p* value (< 0.0001) was highly significant, supporting the rejection of the null hypothesis. The intraclass correlation coefficients (ICCs) for women were 0.06 for the 2‐level model (cluster), 0.10 for the 3‐level model (region), and 0.19 for the 4‐level model (country). The 4‐level model demonstrated the highest ICC, suggesting it was the most suitable model for the analysis.

**TABLE 4:** Summary statistics of the fitted multilevel model for Hb.

Model	DF	Random‐effects parameters				BIC	AICC	ICC
		$\sigma_{ƞ}^{2}$ (country‐level)	$\sigma_{µ}^{2}$ (region‐level)	$\sigma_{\alpha }^{2}$ (cluster‐level)	$\sigma_{\varepsilon }^{2}$ (residual)			
2L.C	26	15.12	—	—	261.24	5,983,732	5,983,434	0.06
3L.R.C	28	11.61	16.41	—	251.02	5,955,703	5,955,381	0.10
4L.CL.R.C	29	11.03	16.67	24.33	226.92	5,921,223	5,920,890	0.19
LR test of *H*_0_: $\sigma_{µ}^{2}$ = 0, *χ* ^2^ = 28,057, *p* value = < 0.0001 (2L.C vs 3L.C.R)								
LR test of *H*_0_: $\sigma_{\alpha }^{2}$ = 0, *χ* ^2^ = 34,493, *p* value = < 0.0001 (3L.C.R vs 4L.C.R.CL)								

*Note:* 2L.C = two‐level model with country‐level random effects. 3L.R.C = three‐level model with region and country random effects. 4L.CL.R.C = four‐level model with cluster, region, and country random effects.

We examined the factors influencing Hb levels, considering clustering effects across four levels. Parameter estimates, along with 95% confidence intervals (CIs), SE, and *p* values, are presented in Table 5. Variables with no noticeable effect were removed from the model. Our multilevel analysis revealed several noteworthy findings. Hb levels decreased by approximately 0.07 g/L for each additional year of age (estimate: −0.07, 95% CI: −0.08 to −0.06, *p* < 0.001). Pregnancy was associated with a significant decrease in Hb levels (estimate: −8.424, *p* < 0.001). Notably, BMI had a modestly beneficial effect on Hb levels. Women whose last menstruation occurred between 6 weeks and 1 year ago exhibited lower Hb levels compared to those with regular menstrual cycles. Conversely, women who were postmenopausal, had undergone hysterectomy, or had never menstruated had significantly higher Hb levels. Additionally, breastfeeding and amenorrhea were associated with lower Hb levels compared to nonbreastfeeding and nonamenorrheic women (both factors *p* < 0.0001). The number of children born per woman was negatively correlated with Hb levels, with each additional child associated with a decrease of 0.145 units (*p* < 0.0001). Socioeconomic status had a substantial positive impact on Hb levels. Middle‐class and rich‐class women exhibited higher Hb levels compared to those from poor‐class families (both *p* < 0.0001). Specifically, middle‐class women had 0.32 units higher Hb levels, while rich‐class women had 0.52 units higher Hb levels. Higher education had a significantly positive effect on Hb levels. Women with higher education levels demonstrated better Hb levels compared to those with no education (estimate: 0.97, 95% CI: 0.76–1.19, *p* < 0.001). Conversely, women with primary and secondary education levels had lower Hb levels. Additionally, literacy was associated with higher Hb levels compared to illiteracy (estimate: 0.33, 95% CI: 0.15–0.50, *p* < 0.001). Married women exhibited higher Hb levels compared to unmarried women (estimate: 0.09, 95% CI: 0.18–0.41, *p* < 0.0001). The use of contraceptive methods, excluding intrauterine devices (IUDs), was positively associated with Hb levels (pill: estimate: 2.78, 95% CI: 2.56–2.99, *p* < 0.0001; other methods: estimate: 0.45, 95% CI: 0.35–0.55, *p* < 0.0001). However, IUD usage was negatively correlated with Hb levels. Tap water (estimate: 0.12, 95% CI: 0.01–0.22, *p* < 0.03) and access to a sanitary toilet (estimate: 0.33, 95% CI: 0.22–0.43, *p* < 0.0001) were both positively associated with Hb levels. Remarkably, access to a sanitary toilet significantly reduced the risk of low Hb levels.

**TABLE 5:** Multilevel modeling for identifying the risk factor of Hb in women aged 15–49 years considering women at level 1, cluster at level 2, the region at level 3, and the country at level 4.

Variables	Estimate	95% CI		SE	*t* value	*p* value
		Lower	Upper			
*Intercept*	112.268	109.113	115.422	1.609	69.76	< 0.0001

*Married women*						
No^∗^						
Yes	0.292	0.178	0.405	0.058	5.04	< 0.0001

*Socioeconomic status*						
Poor^∗^						
Middle	0.320	0.208	0.433	0.057	5.59	< 0.0001
Rich	0.523	0.393	0.653	0.066	7.88	< 0.0001

*Literacy*						
No^∗^						
Yes	0.326	0.154	0.499	0.088	3.70	0.0002

*Highest educational level*						
No education^∗^						
Primary	−0.121	−0.278	0.035	0.080	−1.52	0.1287
Secondary	−0.013	−0.207	0.181	0.099	−0.13	0.8943
Higher	0.969	0.745	1.192	0.114	8.50	< 0.0001

*Currently pregnant*						
No or unsure^∗^						
Yes	−8.424	−8.635	−8.213	0.108	−78.23	< 0.0001

*Currently breastfeeding*						
No^∗^						
Yes	−1.215	−1.332	−1.099	0.059	−20.45	< 0.0001

*Currently amenorrheic*						
No^∗^						
Yes	−0.660	−0.879	−0.441	0.112	−5.91	< 0.0001

*Current contraceptive method*						
Not using^∗^						
Pill	2.777	2.561	2.994	0.110	25.15	< 0.0001
IUD	−1.062	−1.379	−0.745	0.162	−6.56	< 0.0001
Others	0.449	0.351	0.547	0.050	8.96	< 0.0001

*Menstrual period*						
Regular cycle (last menstruation max. 6 weeks ago)^∗^						
Last time 6 weeks to 6 months ago	0.129	0.012	0.245	0.059	2.17	0.0301
Last time to 6 months to 1 year ago	−1.268	−1.507	−1.030	0.121	−10.44	< 0.0001
More than 1 year ago	2.510	2.200	2.820	0.158	15.87	< 0.0001
In menopause/hysterectomy	3.259	3.087	3.431	0.088	37.13	< 0.0001
Before last birth	−0.227	−0.489	0.036	0.134	−1.69	0.0904
Never menstruated	1.530	1.070	1.991	0.235	6.51	< 0.0001

*Tap water*						
No^∗^						
Yes	0.116	0.014	0.219	0.052	2.22	0.0262

*Access to sanitary toilet*						
No^∗^						
Yes	0.328	0.221	0.434	0.054	6.05	< 0.0001

Mother age	−0.069	−0.075	−0.063	0.003	−21.31	< 0.0001

BMI	0.004	0.004	0.004	0.000	75.89	< 0.0001

No. of children born	−0.145	−0.176	−0.114	0.016	−9.09	< 0.0001

^∗^Reference category.

## 4. Discussion

This study represents the first comprehensive investigation into the risk factors affecting women’s Hb levels across South and Southeast Asian countries. We examined how various factors at the individual, cluster, region, and country levels contributed to women’s Hb concentrations. We aimed to explore the risk factors for women’s Hb levels at different levels of clustering, including clusters, regions, and countries, while also considering cluster‐, regional‐, and country‐specific random effects that reflect variations in outcomes across these levels. We were particularly interested in understanding the variations at each level due to the shared environment experienced by women within clusters, regions, and countries. Our findings revealed several important associations. Anemia inversion induces decreased Hb concentration levels during pregnancy, following the physiological changes that occur in this period. This result is also in agreement with previous findings that pregnant women have lower Hb levels which, in turn, results in increased prevalence of anemia [15, 16]. Furthermore, breastfeeding and amenorrheic women were more likely to have lower Hb levels than those who were not. This argument attains credence in terms of categorical nutrition during breastfeeding, wherein breastfeeding mothers should ensure good nutrient intake to the effect of maintaining their health and produce quality mother’s milk [16, 17]. Menstruation is a natural phenomenon; in some cases, it can adversely affect health. Heavy menstrual bleeding can cause a woman to lose large amounts of iron, and consequently, this may lead to decreased Hb concentrations. Our study found that women with their last menstrual period anywhere between 6 months and 1 year ago had lower Hb levels in comparison with those with a regular cycle. These findings are also in agreement with studies stating that long intervening periods between menstrual cycles have been documented to provoke a higher volume of Hb concentrations, most probably related to lesser blood loss during menses [18]. There is a positive correlation between socioeconomic status and the Hb levels. It has been found that the women from middle and higher socioeconomic classes had higher Hb levels than those from poorer levels of living. Similarly, the level of education and literacy also showed a positive association with Hb levels [19–22]. Water supply and sanitary toilets were found to positively influence Hb levels as a result of hygienic conditions with possible effects in limiting parasitic infections [23, 24]. The use of contraception was believed to affect Hb levels; oral pills and other contraceptives were associated with higher levels, while IUDs affected it inversely. This could possibly be associated with different bleeding patterns following the use of various contraceptive methods [25, 26]. This is supported by the higher bleeding and cramping during menstruation associated with copper IUD, and hormonal IUD is recognized to treat irregular or light bleeding that occurs due to iron loss in women [27, 28]. Furthermore, married women had higher Hb levels compared to single women with an increased BMI, which supports data provided in earlier reports [29]. Osborn et al. [30] also noted a positive correlation between married women and Hb levels. Besides, there was a decline in Hb concentration as the age of women and the number of births increased. Older studies have acknowledged these findings [31–33]. Broadly, our study found a large number of categorical variations in Hb concentrations in women at the cluster, regional, and country levels. Such variations might reflect the geographical differences in factors affecting Hb levels. In particular, it points toward the necessity of targeting location‐specific factors in order to mitigate the prevalence of anemia.

## 5. Conclusion

This study highlights that the variations of Hb levels among women aged 15–49 in South and Southeast Asian countries are influenced not only by individual characteristics but also by contextual factors operating at cluster, region, and country levels. Socioeconomic, demographic, and environmental factors were found to be associated with Hb levels in these women. These findings underscore the importance of multilevel public health strategies that address socioeconomic inequalities, improve access to maternal and reproductive health service, and strengthen community‐level health infrastructure. Policymakers should prioritize geographically and socioeconomically disadvantaged populations to reduce anemia‐related disparities. Future research should examine longitudinal patterns and explore broader structural determinants to inform more targeted and sustainable interventions.

## Author Contributions

Abdullah Al Islam: conceptualization; data curation; formal analysis; investigation; methodology; resources; software; validation; visualization; writing–original draft; and writing–review and editing.

Bodrunnahar Barna: methodology; software; validation; visualization; and writing–review and editing.

Md. Amjad Patwary and Mohammad Romel Bhuia: validation; visualization; and writing–review and editing.

Md. Atiqul Islam: conceptualization; methodology; project administration; resources; software; supervision; validation; visualization; writing–original draft; and writing–review and editing.

## Funding

No funding was received for this manuscript.

## Ethics Statement

This study does not involve any human or animal testing.

## Conflicts of Interest

The authors declare no conflicts of interest.

## Supporting Information

Additional supporting information can be found online in the Supporting Information section.

## Supporting information


**Supporting Information** Supporting Table 1: Mean of Hb level by Region of a Country. Supporting Table 2: Descriptive Statistics of socio‐demographic characteristics. Supporting Table 3: Socio‐demographic characteristics by Hb level.

## Data Availability

Upon a reasonable request, data would be made available.
