# Supplementary material for: Socioeconomic, Demographic, and Environmental Determinants of Hemoglobin Levels Among Women: A Multilevel Analysis in South and Southeast Asia
Source: Glob Health Epidemiol Genom. 2026 Jun 7;2026:5430745. doi: 10.1155/ghe3/5430745 (PMC13243770; doi:10.1155/ghe3/5430745)
Supplement: Supplementary file 1 — Supporting Information Supporting Table 1: Mean of Hb level by Region of a Country. Supporting Table 2: Descriptive Statistics of socio‐demographic characteristics. Supporting Table 3: Socio‐demographic characteristics by Hb level. [file GHE3-2026-5430745-s001.docx]

**Supplementary Table 1**: Mean of Hemoglobin(Hb) level by Region of a Country.

| **Country** | **Year** | **Region** | **Mean Hb level (g/L)** |
| --- | --- | --- | --- |
| Bangladesh | 2011 | **-** | **-** |
|  |  | Barisal | 119.6 |
|  |  | Chittagong | 121.9 |
|  |  | Dhaka | 120.7 |
|  |  | Khulna | 122.5 |
|  |  | Rajshahi | 120.8 |
|  |  | Rangpur | 120.1 |
|  |  | Sylhet | 120.5 |
| India | 2015-16 | - | - |
|  |  | Andaman and Nicobar Islands | 112.0 |
|  |  | Andhra Pradesh | 112.9 |
|  |  | Arunachal Pradesh | 123.4 |
|  |  | Assam | 119.3 |
|  |  | Bihar | 114.1 |
|  |  | Chandigarh | 109.0 |
|  |  | Chhattisgarh | 117.9 |
|  |  | Dadra and Nagar Haveli | 108.5 |
|  |  | Daman and Diu | 115.4 |
|  |  | Goa | 123.7 |
|  |  | Gujarat | 114.8 |
|  |  | Haryana | 112.5 |
|  |  | Himachal Pradesh | 120.7 |
|  |  | Jammu and Kashmir | 121.9 |
|  |  | Jharkhand | 112.6 |
|  |  | Karnataka | 118.7 |
|  |  | Kerala | 122.9 |
|  |  | Lakshadweep | 119.0 |
|  |  | Madhya Pradesh | 116.1 |
|  |  | Maharashtra | 118.1 |
|  |  | Manipur | 126.2 |
|  |  | Meghalaya | 116.5 |
|  |  | Mizoram | 127.0 |
|  |  | Nagaland | 127.6 |
|  |  | Delhi | 116.7 |
|  |  | Odisha | 116.6 |
|  |  | Puducherry | 118.4 |
|  |  | Punjab | 117.2 |
|  |  | Rajasthan | 117.9 |
|  |  | Sikkim | 127.1 |
|  |  | Tamil Nadu | 115.4 |
|  |  | Tripura | 116.4 |
|  |  | Uttar Pradesh | 116.5 |
|  |  | Uttarakhand | 121.6 |
|  |  | West Bengal | 113.9 |
|  |  | Telangana | 113.7 |
| **Country** | **Year** | **Region** | **Mean Hb level (g/L)** |
| Maldives | 2016-17 | - | - |
|  |  | Malé | 110.9 |
|  |  | North Region | 117.4 |
|  |  | North Central | 117.5 |
|  |  | Central Region | 110.8 |
|  |  | South Central | 115.1 |
|  |  | South Region | 116.7 |
| Myanmar | 2015-16 | - | - |
|  |  | Kachin | 123.2 |
|  |  | Kayah | 125.6 |
|  |  | Kayin | 120.6 |
|  |  | Chin | 123.9 |
|  |  | Sagaing | 117.9 |
|  |  | Taninthayi | 117.0 |
|  |  | Bago | 119.6 |
|  |  | Magway | 116.5 |
|  |  | Mandalay | 120.1 |
|  |  | Mon | 122.0 |
|  |  | Rakhine | 116.6 |
|  |  | Yangon | 117.5 |
|  |  | Shan | 125.3 |
|  |  | Ayeyarwaddy | 120.8 |
|  |  | NayPyitaw | 120.4 |
| Nepal | 2016 | - | - |
|  |  | Province 1 | 121.6 |
|  |  | Province 2 | 115.6 |
|  |  | Province 3 | 127.5 |
|  |  | Province 4 | 126.8 |
|  |  | Province 5 | 121.1 |
|  |  | Province 6 | 127.5 |
|  |  | Province 7 | 123.2 |

**Supplementary Table 2:** Descriptive Statistics of socio-demographic characteristics.

| **Characteristics** | **Mean ± SD** |
| --- | --- |
| Hb level | 117.5±16.48 |
| Mother age | 29.89±9.76 |
| Body mass index | 2176.1±417.52 |
| No. of children born | 1.89±1.83 |
| Age at first delivery | 20.54±3.84 |
| Number of household numbers | 5.79±2.66 |
| Husband/partner's age | 37.78 ±9.82 |

**Supplementary Table 3:** Socio-demographic characteristics by Hb level.

| **Variable** | **Mean ± SD** | **P-value** |
| --- | --- | --- |
| **Country** |  | <0.0001 |
| Bangladesh | 120.95±13.81 |  |
| India | 117.45±16.54 |  |
| Nepal | 123.05±15.34 |  |
| Maldives | 115.34±13.75 |  |
| Myanmar | 120.25±15.56 |  |
| **Place of residence** |  | <0.0001 |
| Urban | 118.42±16.34 |  |
| Rural | 117.20±16.53 |  |
| **Married women** |  | <0.0001 |
| No | 118.30±16.47 |  |
| Yes | 117.27±16.48 |  |
| **Currently working** |  | <0.0001 |
| No | 117.45±16.76 |  |
| Yes | 118.07±16.84 |  |
| **Socioeconomic status** |  | <0.0001 |
| Poor | 116.24±16.60 |  |
| Middle | 118.04±16.70 |  |
| Richpiped | 118.70±16.134 |  |
| **Literacy** |  | <0.0001 |
| No | 116.11±17.061 |  |
| Yes | 118.23±16.16 |  |
| **Highest educational level** |  | <0.0001 |
| No education | 116.11±17.09 |  |
| Primary | 117.26±16.71 |  |
| Secondary | 118.06±16.25 |  |
| Higher | 119.36±15.32 |  |
| **Currently pregnant** |  | <0.0001 |
| No or unsure | 117.96±16.39 |  |
| Yes | 109.37±16.24 |  |
| **Currently breastfeeding** |  | <0.0001 |
| No | 117.86±16.60 |  |
| Yes | 116.06±15.80 |  |
| **Currently amenorrheic** |  | <0.0001 |
| No | 117.65±16.48 |  |
| Yes | 115.80±16.48 |  |
| **Current contraceptive method** |  | <0.0001 |
| Not using | 117.37±16.62 |  |
| Pill | 120.96±15.49 |  |
| IUD | 118.10±16.98 |  |
| **Variable** | **Mean ± SD** | **P-value** |
| **Menstrual period** |  | <0.0001 |
| Regular cycle (last menstruation max. 6 weeks ago) | 117.95±16.31 |  |
| last time 6 weeks to 6 months ago | 115.99±16.77 |  |
| last time to 6 months to 1 year ago | 112.16±17.30 |  |
| More than 1 year ago | 118.81±16.28 |  |
| In menopause/hysterectomy | 120.0±16.23 |  |
| Before last birth | 115.38±16.60 |  |
| Never menstruated | 117.92±17.53 |  |
| **Currently residing with husband** |  | 0.02 |
| No | 117.09±16.09 |  |
| Yes | 117.28±16.51 |  |
| **Husband/partner's education level** |  | <0.0001 |
| No education | 115.97±17.50 |  |
| Primary | 117.48±16.87 |  |
| Secondary | 117.69±16.85 |  |
| Higher | 118.86±15.91 |  |
| **Husband/partner’s occupation** |  | <0.0001 |
| Manual | 118.78±16.20 |  |
| Non-manual | 117.09±17.04 |  |
| **Tap water** |  | <0.0001 |
| No | 116.80±16.13 |  |
| Yes | 118.58±16.93 |  |
| **Sanitary toilet** |  | <0.0001 |
| No | 115.91±16.58 |  |
| Yes | 118.80±16.30 |  |
